# Compression-Only Cardiopulmonary Resuscitation and Automated External Defibrillator Course for Primary School Students: A Malaysian Pilot Study

**DOI:** 10.3390/children10010058

**Published:** 2022-12-27

**Authors:** Muhamad Nur Fariduddin, Mawarni Mohamed, Mohd Johar Jaafar, Kamarul Baharin, Ching Sin Siau, Kamal Bashah

**Affiliations:** 1 Department of Physical and Health Education, Faculty of Education, Universiti Teknologi MARA (UiTM), Cawangan Selangor, Kampus Puncak Alam, Bandar Puncak Alam 42300, Malaysia; 2 Department of Emergency Medicine, Faculty of Medicine, Universiti Kebangsaan Malaysia (UKM), Kuala Lumpur 56000, Malaysia; 3 Department of Emergency Medicine, Hospital Canselor Tuanku Muhriz (HCTM), Kuala Lumpur 56000, Malaysia; 4 Centre for Community Health Studies, Faculty of Health Sciences, Universiti Kebangsaan Malaysia (UKM), Kuala Lumpur 50300, Malaysia; 5 Abedeen Academy International and Private Schools, Cyberjaya 63000, Malaysia

**Keywords:** kids save lives, cardiopulmonary resuscitation, automated external defibrillator, primary school student

## Abstract

The Malaysian national school curriculum currently lacks resources and tools to enforce CPR education. The aim of this study was to investigate the efficacy of a compression-only cardiopulmonary resuscitation and automated external defibrillator course among primary school students to increase their knowledge and technical skills and improve their attitudes. A quasi-experimental study was conducted using a pre–post non-equivalent design involving 38 students aged 10–12. Cardiopulmonary resuscitation (CPR) and automated external defibrillator (AED) knowledge, technical skills, and attitude towards CPR were assessed in a post test with three-month follow-up. Results of the MANOVA analysis showed significant differences in the level of knowledge (*F* = 10.29, *p* < 0.001) and attitude (*F* = 13.87, *p* < 0.001) based on the students’ age group at the time of the post test. The proportion of students who passed the technical skills component differed significantly by age (χ^2^ = 12.12; *p* = 0.002) and BMI (χ^2^ = 6.34; *p =* 0.041). No significant decay was reported in the total mean scores for knowledge, technical skills, and attitude (*F* = 0.727, *p* = 0.54) at 3-month follow-up. The course helped students perform CPR and utilize AED effectively while promoting a positive attitude with up to 3 months of retention, demonstrating the feasibility of extending the course within the Malaysian primary school curriculum.

## 1. Introduction

Out-of-hospital cardiac arrest (OHCA), which is the third most common cause of death worldwide after cancer and cardiovascular disease [1], is a significant global public health issue, with global incidence and results varying [2,3]. Raising awareness and educating the public about this subject are thus critical. However, studies have revealed that public awareness of CPR differs by region; more than half of adults know little about CPR performance [4]. Studies have predicted that at least 15% of the population is expected to have undergone training to operate public resuscitators that cannot be learned in voluntary courses. Integrating CPR training into school training activities could increase the number of trained resuscitators among the population [5]. The aim of school-based CPR training is to improve bystander CPR and OHCA survival rates [6]. Over time, a school-based CPR intervention could dramatically increase the number of community adults trained in basic life support (BLS) [6,7].

The World Health Organization (WHO) accepted the declaration of “Children Save Lives” created in 2015 by the European Resuscitation Council (ERC), the European Safety Foundation, the International Liaison Committee on Resuscitation, and the World Federation of Anaesthesiologist Societies [5,8], which recommends that CPR instruction be given annually to students in schools worldwide [9]. In addition, in 2011, the American Heart Association (AHA) released an advisory statement suggesting compulsory CPR instruction for schoolchildren [6]. Over the past two decades, many programs were created to teach CPR in classrooms. In addition, in 2011, the AHA proposed a mandatory CPR training with high-quality chest compressions and limited interruptions [6]. Several studies have shown that children as young as nine years old can understand the value of on-going CPR, learn basic life support, maintain their airways, and are fully capable of achieving an adequate chest compression; however, they fail to achieve the AHA and ERC recommended compression depth [7,10]. Since then, there have been debates about school age, physical factors, training factors, retention methods, styles of trainers, and automated external defibrillator (AED) training as predictors of effective high-quality CPR training [10].

In Europe, Kids Save Lives (KSL) proposed that children can perform CPR according to age and physical characteristics and subsequently become potential multipliers of the CPR principles and competencies in their communities. The KSL was recently incorporated in the “Systems Saving Lives” of the ERC Guidelines 2021 [11]. The KSL inspired the world and is changing citizens’ mindset to empower bystanders with CPR competencies through education at all levels. In 2020, CPR training was effectively incorporated into the British national curriculum. The United States has successfully incorporated CPR trainings into secondary and high school curricula In 35 states [12]. Malaysia has more than 10,000 schools, with an estimated 5 million students and 420,000 teachers from preschools to high schools [13]. Over the years, CPR trainings have been offered in Malaysian schools as part of cocurricular activities by professionals including doctors, firefighters, medical students, and paramedics [14].

However, the Malaysian national school curriculum currently lacks certain subjects and tools to enforce CPR [15]. CPR teaching has only been implemented in the Malaysian secondary school curriculum embedded as several topics in a subject called “Pendidikan Kesihatan” (Health Education). In the context of primary schoolchildren, there is no CPR teaching conducted during the school period. Moreover, no specific course or handbook has been designed to address this topic, failing to fulfil the requirement of the WHO policy [8], which recommends at least two hours of annual training, including theory and practical training, at all levels of education. To address this limitation, in this study, we developed a compression-only cardiopulmonary resuscitation and automated external defibrillator course for primary school students. The first objective was to assess the preliminary efficacy of the course on primary school students’ knowledge, technical skills, and attitudes and, secondly, to measure the retention rate of the students 3 months following the course.

## 2. Materials and Methods

### 2.1. Study Design and Participants

This quasi-experimental study was conducted using a pre–post non-equivalent dependent variable (NEVD) during the academic year of 2022 (1 April to 31 August). Students aged 10–12 years old from a Malaysian private school were the candidates selected to participate in the study. Using a priori analysis (G*Power Software version 3.9.1) with α = 0.05, *p* = 0.80, and medium effect size (0.50), a total of 36 students was estimated with a 5% attrition rate, with each group being represented by 12 students from each of years 4, 5, and 6. Non-Malaysian children, those with a physical handicap or underlying diseases that significantly limited their physical performance, non-fluent (unable to speak, write, or understand Bahasa Malaysia), and those whose parents did not consent were excluded.

### 2.2. Ethics

The study protocol was approved by the research ethics committee of Universiti Teknologi MARA (UiTM) from November 2021 until June 2022 with ethical approval number REC/11/2021(MR/886). We obtained written informed consent for the study from the school principal and parents.

### 2.3. Course Content

**Figure 1:**
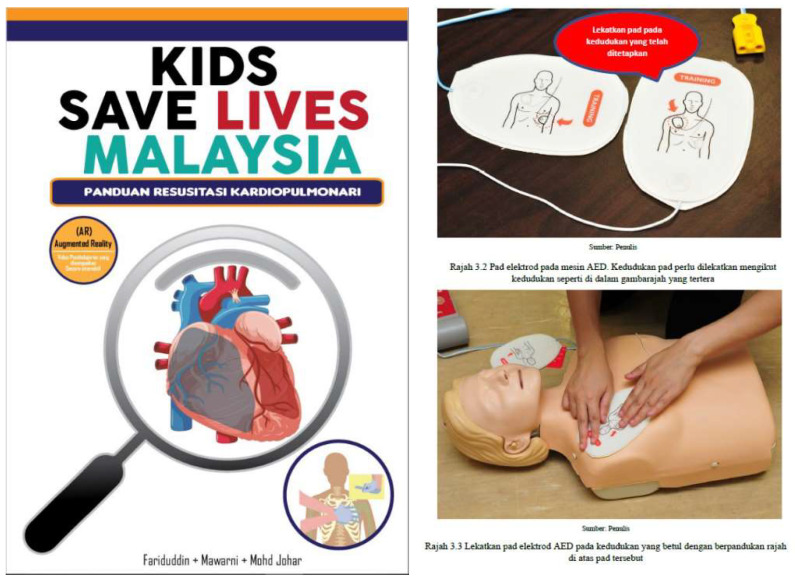
KSLM Handbook.

We recruited three instructors for the course for each group (years 4, 5, and 6). The instructors were certified American Heart Association (AHA) instructors (physicians, paramedics, and health educationists) with varying BLS teaching experience. We developed a handbook called KSLM—Panduan Resusitasi Kardiopulmonari (National Language) in the Malay language that included lesson plans (compression-only CPR and usage of AED), a knowledge test, a practical skills testing checklist, and teaching videos, which were incorporated via the application of virtual reality (VR) (Figure 1). The content of this handbook was adapted from the AHA 2020 Heartsaver programme, and the handbook, which had an excellent content validity index (CVI), was validated by six experts from various fields [16]. Throughout the course, we used the Laerdal QCPR Manikin and Laerdal AED Training.

### 2.4. Implementation of the Course

**Figure 2:**
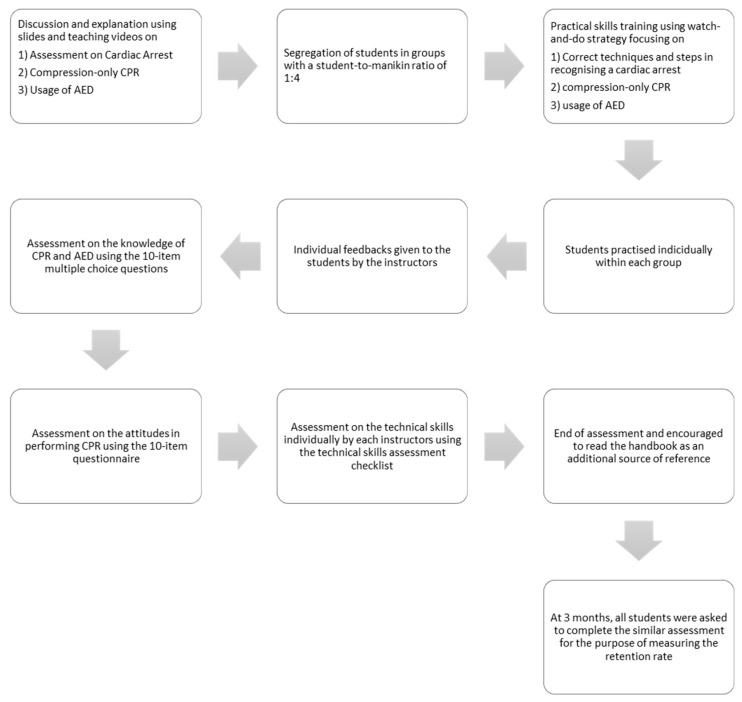
An overview of the course and assessment.

The initial course was led by the instructors through discussion and explanation based on a slide presentation, together with the teaching videos focusing on identifying a cardiac arrest victim and initial steps taken in addressing a cardiac arrest. This was followed by the next topic, which was compression-only CPR and the usage of AED. Teaching videos were used as part of the demonstration. In total, each instructor spent approximately an hour on the initial part. The students then practised compression-only CPR and AED skills on manikins (90 min). We adopted a watch-and-do strategy. First, the students were trained in groups with a student-to-manikin ratio of 1:4. Next, the instructors demonstrated the correct techniques and steps in recognising a cardiac arrest victim, performing high-quality chest compression followed by the steps to use the AED. The students then practised each of these techniques and steps individually within each group, and specific feedback was given to the individual students as they practised (Figure 2).

### 2.5. Technical Skills Assessment

Towards the end of the course, the students were individually assessed on their ability to act in the event of a cardiac arrest in a school setting. The instructor assessed the students’ technical skills individually; each student was individually assessed in a room not visible to other students during the event. The scoring was based on the AHA Heartsaver course, comprising 10 items as follows: (1) setting a safe scene, (2) response of patient, (3) calling for help, (4) performing high-quality compressions, (5) turning on the AED, (6) attaching AED pads, (7) analysing heart rhythm, (8) following AED instructions, (9) pressing a button to deliver shock, and (10) resuming chest compression following shock. Each checklist item was evaluated with a “YES” or “NO”. A pass was given if all the items were marked as “YES”. To measure skill retention, a similar scenario was presented to the students 3 months following the initial course. By the end of the assessment, each student was encouraged to read the handbook provided and to utilise the VR application as an additional source of reference (Figure 2).

### 2.6. Knowledge of CPR and AED

The students completed validated 10-item multiple choice questions (MCQs) at the end of the course and subsequently at 3 months later for the post-test follow-up. The MCQs were based on information taken from the KSLM handbook and previously validated by selected professionals on their face, content, construct, and criterion validity and tested for their reliability [16]. An MCQ passing mark of 90% was adopted for this study, consistent with the official AHA guidelines. One mark was awarded for each correct answer, whereas no penalty was given for incorrect answers (Figure 2).

### 2.7. Attitudes in Performing CPR

A validated questionnaire consisting of 10 statements about e attitudes in performing CPR was given to the students following the completion of the MCQ test [17]. Their attitudes were measured using 5-point Likert scale responses ranging from 1 (strongly disagree) to 5 (strongly agree). The responses to the 10 attitude statements were summated to give an overall attitude score, ranging from 10 (lowest) to 50 (highest), with higher scores representing a more positive attitude towards performing CPR. The students were asked to complete the same questionnaire 3 months after the course during the follow-up assessment (Figure 2).

### 2.8. Statistical Analysis

The demographic data were reviewed, organised, tabulated, and statistically analysed using descriptive statistics. The MCQ knowledge, technical skills, and attitude scores were reported as frequency counts (%) and post-course mean at 3-month follow-up. Following the course, we used a chi-square test of independence to analyse the association between knowledge, body mass index (BMI), gender, and age towards technical skills and a one-way multivariate analysis of variance (MANOVA) to compare the students’ knowledge, technical skills, and attitude towards performing CPR based on their age. To measure the effect of retention after 3 months, the mean MCQ knowledge, technical skills, and attitude scores were compared using a one-way multivariate analysis of covariance (MANCOVA). For both MANOVA and MANCOVA analyses, univariate normality was assumed via Shapiro–Wilk tests and box plots. No multivariate outliers were discovered in the data, confirming multivariate normalcy. The absence of excessive correlations between the dependent variables indicated that multicollinearity was not an issue. Additionally, the relationship of the dependent variables was linear. Finally, at *a* = 0.001, Box’s M was not significant, showing that variance–covariance matrices were homogeneous. SPSS V.27.0 (IBM) software was used for data analyses [18]. The level of significance was set at *p* < 0.05.

## 3. Results

A total of 38 students (22 boys and 16 girls) were included and grouped, and they attended the course. Their demographic profiles are summarised in Table 1. There was no loss to follow-up 3 months after training.

### 3.1. Knowledge, Technical Skills, and Attitude towards Performing CPR

The percentage of students who answered at least 9 questions (out of 10) reduced from post-course to 3-month follow-up in year 4 (7.9% vs. 5.25%), whereas there was an increase in year 5 (2.6% vs. 5.25%). None of the students from year 6 passed the knowledge test. As for the technical skills (scores of 90% and above), the questions answered by year 4 (18.5%) showed a reduction after 3 months (2.6%), with no changes in year 6, and none of the year 5 students passed the technical skills assessment. Reductions in mean attitude scores among year 4 (*M* = 38.07 vs. *M* = 33.83) and year 5 (*M* = 39.43 vs. *M* = 35.00) students were noted, and there was an increase in year 6 (*M* = 29.72 vs. *M* = 33.21). Overall, the total mean scores for knowledge (*M* = 6.34 vs. *M* = 6.05), technical skills (*M* = 8.02 vs. *M* = 6.96), and attitude (*M* = 35.74 vs. *M* = 34.01) showed a reduction from post-course to 3-month reassessment (Table 2).

### 3.2. Students’ Knowledge, Technical Skills, and Attitude toward Performing CPR Following the Course

A MANOVA was used to compare the students’ knowledge, technical skills, and attitude towards performing CPR based on their age. There was a significant difference in the level of knowledge, technical skills, and attitude among year 4–6 students (*F* = 10.59, *p* < 0.001). At a Bonferroni corrected alpha level of 0.017, a significant difference was reported with respect to technical skills (*F* = 10.29, *p* < 0.001), with year 4 recording the highest mean scores, followed by years 6 and 5 (*M* = 8.93 vs. *M* = 8.40 vs. *M* = 6.74), and attitude (*F* = 13.87, *p* < 0.001), with year 5 scoring the highest, followed by years 4 and 6 (*M* = 39.43 vs. *M* = 38.07 vs. *M* = 29.72, respectively) (Table 3).

### 3.3. Relationship between Knowledge, BMI, Age, and Gender with Respect to Technical Skills

We employed Pearson’s chi-square test of independence to evaluate the association of pass/fail with respect to technical skills with knowledge, BMI, age, and gender following the course among the students. The chi-square test was statistically significant only in the age χ^2^ = 12.12; *p* = 0.002 with age 10 (n = 7) and age 12 (n = 1) students and BMI χ^2^ = 6.34; *p* = 0.041 with overweight (n = 4), normal (n = 2), and underweight (n = 2) students who passed the technical skills test (Table 4).

### 3.4. Course Retention Effects

The means at 3 months for all variables were analysed with MANCOVA to assess the differences between the age groups with the total mean scores from the post course as a covariate. The findings confirmed that there was no significant decay of the variables measured among the students from age 10 to 12 years (F = 0.727, *p* = 0.54). Despite the lack of a significant effect, year 5 exhibited retention of knowledge (M = 6.42, *p* = 0.48), and year 6 retained most of the technical skills (M = 7.76–7.23, *p* = 0.89), followed by year 5 (M = 37.65–35.00, *p* = 0.85) for attitude (Table 5).

## 4. Discussion

The results of this study demonstrate that brief and simple training delivered during school hours by selected healthcare professionals as instructors enabled students in years 4–6 (10–12 years old) to activate the chain of survival, initiate the basics life support, and utilise an AED machine to perform high-quality CPR, which was feasible and widely accepted by students and school administrators. With a nearly 20% passing rate on the knowledge and technical skills following the short course, together with a positive attitude towards performing CPR, we found that the students were able to understand the importance of bystander CPR and were able to learn how to perform basic CPR, retaining this knowledge after 3 months [7,12,20]. A study on 4–5-year-old children found that they were able to assess breathing and consciousness, contact the emergency number, provide sufficient information on the phone, place the victim into the recovery position, and open the airway when tested after 2 months of training [21]. With a similar assessment as that mentioned above, the performance of 6–7-year-old children was found to be excellent, and for CPR, it was deemed reasonable to good [22]. Furthermore, in this study, we found that several students were able to obtain perfect scores on the knowledge test, passed the technical skills assessment, and obtained a good attitude score. This showed that the learning outcomes of the course were met for the students, which further justifies teaching BLS to schoolchildren without waiting for them to reach adolescence or adulthood.

When the effectiveness of the course for students of different ages was assessed, nearly all students were able to acquire the knowledge, as demonstrated by average scores of 60%, with 10.5% passing the test and substantially retaining the knowledge at the 3-month follow-up. These results were the result of the knowledge gained after watching the training videos, together with the hands-on practice delivered during the course. These results are also comparable to those reported in several other studies [23,24]. For the assessment of technical skills, average scores exceeding 70% were achieved by students in the 10- and 12-year-old groups. Despite not being able to perform the full sequence with every step in the correct order, especially effective chest compressions, it is fair to say that they exhibited good knowledge acquisition, as reflected in the data and those reported in past studies [25,26]. Several studies have shown that children as young as nine years old can understand the value of ongoing CPR, learn basic life support, maintain their airways, and are fully capable of achieving an adequate chest compression; however, they fail to achieve the AHA and ERC recommended compression depth [27,28]. In addition, an effective chest compression requires minimum physical strength and endurance that may be out of the range of a normal schoolchild; studies have addressed the cut-off point of age that is acceptable, with an age of at least 13 years considered necessary to achieve an effective compression with the required depth [29,30,31]. Carrying out the steps consecutively in adherence to the AHA guidelines is no simple task, even for an adult who has attended the standard BLS training within the stipulated time frame given [32]. With respect to attitude, a positive attitude towards CPR predicts a person’s willingness to perform CPR during an emergency [33]. In this study, the total mean scores for attitude were above 70%, reflects good attitudes. During the course, the healthcare professionals’ feedback was that most students were self-directed, motivated, and willing to learn the course material. Furthermore, no significant decay was reported in terms of attitude scores 3 months following the course, indicating that the course improved confidence levels in performing CPR and intentions to perform CPR.

Previous studies suggested that the main factor affecting the quality CPR was an effective and high-quality chest compression [34,35,36,37]. The ERC and AHA guidelines suggest a chest compression rate of 100–120 per minute with a depth of 50–60 mm [38]. Therefore, we investigated the association of potential barriers that may affect the quality of chest compression and found that the students’ knowledge and gender did not affect the quality of chest compression. Instead, effective chest compression was associated with body weight; students who were underweight or students weighing less than 100 lbs had higher failure rates compared to overweight students [39]. As described in previous studies, physical characteristics including body weight and height are important factors contributing to effective chest compression, which is a main component of delivering high-quality CPR. The development of these characteristics is strongly associated with anthropometric growth. In a study investigating the variability in compression depth among three age groups (12–14, 14–16, and 16–18 years old), the 12–14-year age group achieved 23%, with 80% in the 14–16-year and 87% in the 16–18-year groups. A significant positive correlation was found between CPR and physical variables, especially weight >100 lbs [40]. Another study reported that, with respect to the age of the participants, achieving the recommended depth of compression is a nearly impossible goal for children under 13 years old [41]. However, this was not the case in this study, as the 10-year-old students in the underweight to normal body weight range achieved the highest passing rate for the technical skills assessment.

In this study, we also speculated that other factors, such as prior physical training, engagement in the task, peer influence, and support, might be involved in this age range. A possible explanation is that the students in this age range were strong enough to compress the chest to an adequate depth on an adult-sized manikin. This finding was also reported in a comparable study involving 721 schoolchildren (10–12 years old), with 7–9.4% of them successfully achieving a correct compression depth and 21.8–22.8% of them obtaining a correct compression rate, suggesting that it is not impossible for students below the age of 13 to achieve a successful rate of an effective chest compression as per the adult [42]. However, of the many technical skills examined, the ability to perform a correct compression rate and depth was the most difficult for a large portion of the students in this study, aside from the other compression parameters that do not depend on weight and height, such as correct hand positioning [25,26,42]. When it comes to compression rate, the factors at play are not only anthropometric characteristics or the ability to assimilate knowledge but also the level of motor skill development and maturity. The mastery of fundamental motor skills is strongly related to physical activity in children and adolescents [43] and may contribute to physical, social, and cognitive development. Mastering fundamental motor skills also is critical to fostering physical activity because these skills serve as the foundation for more advanced and specific movements [43,44]. Motor development depends on the interaction of experience (e.g., practice, instruction, and appropriate equipment) with an individual’s physical, cognitive, and psychosocial status and proceeds predictably across developmental periods.

It is also important to be mindful of the wide interindividual variation in the rate at which children develop motor skills, which is determined by their biological makeup, their rate of physical maturation, the extent and quality of their movement experiences, and their family and community environment. This development determines the ability to move at a given pace. Children who do not acquire fundamental motor skills will likely have trouble transitioning their movement and developing the ability to move at a pace [45]. An effective chest compression requires the ability to compress with a depth of 2 inches together with a compression rate of 100–120 compressions per minute. In this study, the possibilities of having a group of children with advanced motor skill development and maturity may contribute to the high passing rate in younger children yielding the variations between passed and failed among the students with respect to technical skills [46].

At the 3-month follow-up, the students failed to maintain a high level of knowledge, technical skills, and attitude. A possible explanation is that the quality of the students’ knowledge and CPR skills significantly decreased, a finding that is consistent with those reported in other studies [47,48]. This discrepancy may be explained by the differences in the training duration. Several studies have reported that a relatively short duration of CPR training using hands-on practice was effective for the acquisition and retention of knowledge [47,48]. In contrast, other studies have reported longer durations of hands-on practice to improve the acquisition of CPR skills after training [49], suggesting that although hands-on CPR may be easier and shorter in duration to learn and perform, it is difficult to maintain students’ skills and knowledge over time. Several strategies including short and frequent refresher training or reinforcement used throughout the school year can be explored to provide an effective retention method.

The results of this study imply that children aged 10–12 years old were physically fit to perform effective CPR and use AED correctly up to several months after the course. This finding is consistent with a wide range of BLS programmes in schoolchildren [10,37,50]. Despite the finding that a majority of the students was not able to perform an effective chest compression, they had acquired the knowledge and skills to perform effective and high-quality CPR. The study results also show that school is an ideal setting for CPR training to have a significant impact on the response to OHCA, thus supporting the inclusion of the course in the Malaysian school curriculum. This would be consistent with the mandatory BLS training in the national curriculum in the UK, China, and the United States. Teaching students at a young age provides knowledge for use when they are older. Furthermore, by starting early, a revision is possible at the school with the prospect of improved knowledge, skills attainment, and retention.

The main strength of this study is the usage of CPR knowledge questions adapted from the AHA and a survey questionnaire with validated properties. In addition, technical skills were assessed using a QCPR manikin (Laerdal) and marked by a certified AHA instructors using the AHA CPR and AED checklist for the Heartsaver programme. The course was designed by the selected experts and was previously validated for its content properties. However, the present study is also subject to several limitations. First, the students may have had difficulties in truly understanding the delivery of the course in Malay language, as the school’s environment and modes of communication were in English. For example, the students faced difficulties in interpreting the basic terminology in Malay language while answering the survey, the MCQs test, and during class interaction. Secondly, the number of subjects recruited for this study was relatively low compared to other studies. As this was a pilot study and given the small sample size, the inferences drawn based on age differences should be interpreted with caution. Thirdly, the instructor-to-student ratio of 1:4 for manikin practice may pose difficulties in recruiting enough healthcare professionals to teach these courses on a long-term basis. Finally, in this study, we only measured the retention rate at 3-month follow-up instead of a continuous assessment throughout the school year. In improving the validity of the course implementation and content, an annual review will be conducted with the respective students and schoolteachers to gather feedback for further improvement. A train-of-trainer session will also be conducted with the respective facilitators to improve the delivery and assessment of the course content. Therefore, the results of this study could inform future designs to implement the course on a larger scale in Malaysian government schools across the country.

## 5. Conclusions

The brief Compression-Only Cardiopulmonary Resuscitation and Automated External Defibrillator course, which includes theoretical and practical trainings by healthcare professionals, allowed primary school students to learn how to identify an emergency, initiate the chain of survival, perform effective and high-quality CPR, use an AED, and promote a positive attitude in performing CPR with retention up to 3-months following the training. We recommend that this study be scaled up to deliver the course in other private and national primary schools in Malaysia, together with the additional reassessment up to 12 months within the school year to obtain data on the decay pattern. Moreover, larger-scale data can also be collected from across Malaysia before implementing it as part of the Malaysian curriculum.

## Figures and Tables

**Table 1:** Demographic profile.

Demographics	Frequency (%)
Gender Male Female	22 (57.9) 16 (42.1)
Ethnicity Malay Chinese Others	36 (94.7) 1 (2.6) 1 (2.6)
Religion Muslim Christian	37 (97.4) 1 (9.6)
Age and Year 10 (Year 4) 11 (Year 5) 12 (Year 6)	14 (36.8) 19 (50) 5 (13.2)
Body Mass Index (BMI) [19] Underweight Normal Overweight	7 (18.4) 19 (50) 5 (13.2)

**Table 2:** Mean scores and success rate of knowledge, technical skills, and attitude towards CPR post course and after 3 months.

Variable	Age	Post-Course			After 3 Months		
		Mean	Result (Frequency, %)		Mean	Result (Frequency, %)	
			Pass	Fail		Pass	Fail
Knowledge	10 11 12	6.64 6.37 6.00	3 (7.9) 1 (2.6) -	- 18 (76.3) 5 (13.2)	5.47 6.42 6.25	2 (5.25) 2 (5.25) -	12 (31.58) 17 (44.7) 5 (13.2)
	Total	6.34	4 (10.5)	34 (89.5)	6.05	4 (10.5)	34 (89.48)
Technical Skills	10 11 12	8.93 6.74 8.40	7 (18.5) - 1 (2.6)	7 (18.4) 19 (50) 4 (10.5)	6.75 6.90 7.23	1 (2.6) - 1 (2.6)	13 (34.24) 19 (50) 4 (10.5)
	Total	8.02	8 (21.1)	30 (78.9)	6.96	2 (5.26)	36 (94.74)
Attitude	10 11 12	38.07 39.43 29.72			33.83 35.00 33.21		
	Total	35.74			34.01		

**Table 3:** Multivariate test of knowledge, technical skills, and attitude towards CPR among students following the course.

Source	Value	*F*	Hypothesis *df*	Error *df*	Sig	*η* ^2^
Pillai’s Trace	0.135	10.59	3	34	<0.001 *	0.483
Source	Dependent Variable	*df*	Mean Square	*F*	Sig	η^2^
Students’ Age	Technical Skills Score Attitude Score	2 2	11.83 16.79	10.29 13.87	<0.001 * <0.001 *	0.370 0.442

Sig = significance; * significant at the level of *p* < 0.001.

**Table 4:** Association between knowledge, BMI, age, and gender with respect to technical skills following the course.

Variables		Technical Skills		Chi-Square Value *p* Value	
		Pass	Fail		
Knowledge	Pass Fail	1 7	3 27	0.042	0.838
BMI	Underweight Normal Overweight	2 2 4	5 21 4	6.348	0.041 *
Age	10 11 12	7 0 1	7 19 4	12.12	0.002*
Gender	Male Female	5 3	17 13	0.088	0.767

* Significant at the level of *p* < 0.05.

**Table 5:** Estimated marginal means of knowledge, technical skills, and attitude towards CPR among students 3 months following the course.

Variable	Age	Baseline Mean (Covariate)	Post-3-Month Mean
Knowledge	10 11 12	6.42	5.47 6.42 6.25
Technical Skills	10 11 12	7.76	6.75 6.90 7.23
Attitude	10 11 12	37.65	33.83 35.00 33.21

## Data Availability

The data presented in this study are available upon request from the corresponding author. The data are not publicly available due to confidentiality.
